# *Trichoderma hamatum* Strain Th23 Promotes Tomato Growth and Induces Systemic Resistance against Tobacco Mosaic Virus

**DOI:** 10.3390/jof8030228

**Published:** 2022-02-25

**Authors:** Ahmed Abdelkhalek, Abdulaziz A. Al-Askar, Amr A. Arishi, Said I. Behiry

**Affiliations:** 1 Plant Protection and Biomolecular Diagnosis Department, ALCRI, City of Scientific Research and Technological Applications, New Borg El Arab City 21934, Egypt; 2 Department of Botany and Microbiology, College of Science, King Saud University, P.O. Box 2455, Riyadh 11451, Saudi Arabia; 3 School of Molecular Sciences, The University of Western Australia, Perth, WA 6009, Australia; 22650755@student.uwa.edu.au; 4 Agricultural Botany Department, Faculty of Agriculture (Saba Basha), Alexandria University, Alexandria 21531, Egypt; said.behiry@alexu.edu.eg

**Keywords:** *Trichoderma*, TMV, enzymes, stress markers, pathogenesis-related proteins, molecular mechanism, tomato

## Abstract

*Trichoderma hamatum* strain Th23, isolated from tomato roots, was molecularly identified using phylogenetic analysis based on *ITS*, *tef1*, and *rpb2* gene sequences and evaluated for its efficiency in suppressing tobacco mosaic virus (TMV) infection for the first time. Under greenhouse conditions, the application of Th23 promoted tomato growth with significant increases in shoot and root parameters as well as improved total chlorophyll content. Compared to the nontreated tomato plants, the soil pretreatment of tomato plants 48 h before TMV inoculation produced a significant reduction in the TMV accumulation level by 84.69% and enhanced different growth parameters. In contrast, TMV had a deleterious impact on fresh and dry matter accumulation and inhibited photosynthetic capacity. Furthermore, the protective activity of Th23 was associated with a significant increase in reactive oxygen species scavenging enzymes (PPO, CAT, and SOD) as well as decreased nonenzymatic oxidative stress markers (H_2_O_2_ and MDA) compared to the TMV treatment at 15 days post-viral inoculation (dpi). In addition, considerable increases in the transcriptional levels of polyphenolic genes (*HQT* and *CHS*) and pathogenesis-related proteins (*PR-1* and *PR-7*) were shown to induce systemic resistance against TMV. Consequently, the ability of *T. hamatum* strain Th23 to promote plant growth, induce systemic resistance, and boost innate immunity against TMV infestation supported the incorporation of Th23 as a potential biocontrol agent for managing plant viral infections. To the best of our knowledge, this is the first report of the antiviral activity of *T. hamatum* against plant viral infection.

## 1. Introduction

Tobacco mosaic virus (TMV) is a single-stranded positive-sense RNA belonging to the Tobamovirus genus that infects many plant species in several families, primarily tobacco and tomato plants as well as most Solanaceae family plants [1,2]. TMV infection results in mosaic signs on the leaves, yellowing plant tissue, and significant economic losses worldwide [3]. Since TMV has an extensive host range, it has a devastating effect on the hosts’ yields. New approaches to controlling TMV are required because of the shortage of suitable and effective control [4]. Biological control agents have long been shown to improve plant defense systems and lower disease severity and incidence. Among the biological control agents that have proven to have a reasonable degree of pathogen control, plant growth-promoting fungi (PGPF) are well known for their ability to minimize the disease incidence of many fungal, bacterial, and viral plant pathogens and trigger plant defense reactions [5]. The use of beneficial Trichoderma fungi to prevent viral plant diseases has attracted interest because it is a safe and environmentally friendly method of controlling pathogens [6].

*Trichoderma* spp. are free-living parasitized fungi known as bioagents because of their competency to suppress or even kill phytopathogens [7,8]. They can also elicit a host immune response against pathogenic microbes with potential benefits for vegetative growth [9] and enhance photosynthetic rates and respiration reactions by reconfiguring plant gene transcription [10]. In tomatoes, *Trichoderma hamatum* successfully induced resistance to *Botrytis cinerea* and *Xanthomonas vesicatoria* [11,12]. Mastouri et al. [13] found that treating tomato seeds with *T. harzianum* relieved various biotic and abiotic stressors. Thus, after colonizing roots, *Trichoderma* species can interact well with plants, and in some cases, chemically operate as endophytic symbiotic organisms. As a result, they can modify the activation of many plant genes and even plant physiology [14]. Through studying several molecular and biochemical components of host–virus interaction and establishing the precise role of ROS, it was reported that *T. harzianum* had positive effects on the tomato defense system against cucumber mosaic virus (CMV) infection [15].

Plants generally compensate for diseases through a variety of cellular processes including (i) up- or downregulation of certain genes; (ii) changes in the levels of different compounds known to play a role in the host defense pathway including reactive oxygen species (ROS); (iii) increased expression of particular transcriptional regulators, defensive scheme genes, and heat-shock proteins; and (iv) improvement in the mobility of biomolecules, enzymes, and growth factors involved [16,17]. Pathogenesis-related proteins (PRs) are primarily found in the systemic acquired resistance (SAR) pathway and are potent as antipathogenic agents [18]. Furthermore, Singh et al. [19] found that biotic inducers raised the levels of several PRs such as peroxidase and chitinase isozymes. The increase in *PR-1* as a SA molecular marker gene is closely linked to the activation of salicylic acid (SA) during pathogen infection and a variety of physiological responses in plants in response to biotic stressors [20,21]. Polyphenolic substances are among the secondary metabolites that play a significant role in plant development, growth, and tolerance to biotic and abiotic stresses [22]. The biochemical and molecular research on the host–pathogen–antagonist relationship is critical for understanding the dynamics of infectious diseases. By penetrating the plant epidermal tissues, *Trichoderma* spp. has the capability to colonize plant roots, which usually results in triggering different metabolic pathways by modifying gene expression [23,24]. It has been observed that when a plant comes into contact with a pathogen, a SAR mechanism is triggered, but when it comes into contact with a nonpathogenic organism, an ISR mechanism is activated [25,26]. Many studies have used transcriptional analysis to determine the expression of defense genes and proteins in response to *Trichoderma*-induced resistance (TIR) against various pathogens, which has established that TIR is preceded by the upregulation of genes encoding vital defense enzymes and PR-proteins [27,28]. *T. hamatum* UoM13 was reported as a mediator of plant systemic immunity by significantly increasing the activity of SA-inducible genes such as glucanase, *PPO*, *POX*, *PAL*, *PR-1*, and *PR-5*, all of which are considered essential SAR markers [27].

Although there is already evidence that *Trichoderma* spp. has a role in viral disease resistance in plants, there have been no previous studies exploring alterations in plant physiology and antiviral activities of *T. hamatum* against plant viral infections. The primary goal of this investigation was to isolate, identify, and evaluate the protective activity of the *T. hamatum* strain Th23 against TMV infection under controlled greenhouse conditions for the first time. Moreover, the effects of Th23 on the viral symptom appearance, tomato growth parameters, chlorophyll content, and the accumulation level of TMV inside infected tissues were estimated. Furthermore, the transcriptional level changes of two polyphenolic genes, *hydroxycinnamoyl Co A quinate hydroxycinnamoyl transferase* (*HQT*) and *chalcone synthase* (*CHS*), and three PR proteins (*PR-1*, *PR-2*, and *PR-7*) as well as some defense enzymes including polyphenol oxidase (PPO), catalase (CAT), and superoxide dismutase (SOD) were evaluated. The ability to use Th23 against TMV infection could be crucial for developing a thorough understanding of the plant–pathogen–bioagent complex and building effective viral disease control strategies.

## 2. Materials and Methods

### 2.1. Collecting, Isolating, and Identifying Samples

*Trichoderma* isolate was obtained from soil rhizosphere samples collected from tomato-growing areas in Egypt’s El-Behira governorate. Using the *Trichoderma* TSM specific media, the antagonistic isolate was isolated using the serial dilution plate method by Elad et al. [29]. The resultant fungal colonies were purified using the hyphal tip separation procedure, and the fungus was kept on potato dextrose agar (PDA) (HiMedia Laboratories Pvt. Limited, Mumbai, India) for further examination. To identify the isolate, the *ITS, rpb2*, and *tef1* genes (Table 1) were used as well as the morphological traits. In PCRs, a 1 µL volume of each primer pair (10 pmole), 20 µL of 2x MyTaq Red Mix (Bioline Inc., Ansan, Korea), 2 µL of fungus DNA, and 26 µL of molecular grade water were used. Cycling was performed as follows in a gradient SureCycler (Agilent Technologies, Santa Clara, CA, USA): an initial step at 94 °C for 5 min, followed by 40 cycles at 95 °C, 55 °C, and 72 °C each for 1 min, and a final extension step at 72 °C. After sequencing the PCR amplicons, the nucleotide sequences were aligned using MEGA X software. The resulting sequences were compared to those in the GenBank database using the NCBI-BLAST tool.

### 2.2. TMV Isolate, Inoculum Preparation, and Greenhouse Investigation

The tobacco mosaic virus (TMV) strain KH1 (MG264131) used in this study has been previously characterized [37]. To prepare 20 μg/mL of TMV inoculum, the concentration was adjusted with 100 mM phosphate buffer, pH 7. The tomato virus-free Carmen F1 seeds (Nongwoo Bio, Co. Ltd., Gyunggi-do, Suwon, Korea) were sown in a controlled greenhouse in trays prefilled with peat moss mixture. After three weeks, the seedlings were transported into 25 cm pots, and each pot was filled with five kilograms (kg) of sand and clay (1:1). Trichoderma inoculum was prepared by serial dilution and used as 5 mL/kg at a 1 × 10^8^ spores/mL concentration. A week later, the two upper leaves of each tomato seedling were dusted with carborundum and gently mechanically inoculated with 1 mL of semi-purified TMV [38].

There were four treatments in the trial; each treatment consisted of three replicates, and every replicate had a three-pot. The tomato plants treated with TMV-free inoculum buffer and foliar sprayed with sterile water served as the control (mock treatment). Plants mechanically inoculated with TMV only were used as the TMV treatment. Plants inoculated with *Trichoderma hamatum* only were identified as the Th23 treatment. Plants were inoculated with *T. hamatum* 48 h before being mechanically inoculated with TMV (Th23 + TMV treatment). All of the plants were kept in the greenhouse at a temperature of 28 ± 2 °C/18 ± 2 °C at day/night and 75% relative humidity. The TMV symptoms were observed, day after day. At 15 days post-viral inoculation (dpi), nine leaves collected from three plants per pot were combined and ground for total chlorophyll content quantification by a SPAD-502Plus meter (Konica Minolta, Inc., Tokyo, Japan) as well as the enzyme activity estimations and RNA extraction. The plant’s length and fresh and dried weights of each treatment’s shoot and root systems (g) were assessed. 

### 2.3. Determination of Enzyme Activity

All of the fine chemicals used in this section were purchased from Sigma-Aldrich (St. Louis, MO, USA) and all measurements were performed by a UV–Vis spectrophotometer (EMCLAB Instruments GmbH, Duisburg, Germany).

#### 2.3.1. Oxidative Stress Markers

##### Malondialdehyde

The thiobarbituric acid (TBA) method [39] was used to determine the malondialdehyde (MDA) content in plant leaves. The leaves were pulverized with 0.1% trichloroacetic acid (TCA) and centrifuged at 12,000× *g* for 30 min. After that, the supernatants were incubated with a mixture of TCA and TBA (4:1 *v/v*) for 30 min. Then, all the mixes were quickly chilled. The MDA content was measured at 600 nm and expressed as μM/g fresh weight (FW).

##### Hydrogen Peroxide

To determine the amount of hydrogen peroxide (H_2_O_2_) in tomato leaves, the samples were homogenized with 0.1% TCA [40]. Equal amounts of the supernatant, 10 mM of potassium dihydrogen phosphate (pH 7), and potassium iodide (1 M) were mixed and left at 25 °C for 15 min., then centrifuged at 12,000× *g* for 10 min. The resultant supernatant absorbance was measured at 390 nm. The quantity of H_2_O_2_ was expressed as μM/g FW.

#### 2.3.2. Antioxidant Enzymes

The extracts used in this section were obtained by pulverizing tomato leaves in phosphate buffer and spun at 12,000× *g* for 30 min. The pellets were discarded, and the extracts were preserved at −20 °C until used.

##### Polyphenol Oxidase

The polyphenol oxidase (PPO) enzyme was determined using a mixture of enzyme extract, Tris-HCl (50 mM, pH 6), and quinone (1:2:1, *v/v/v*), then left at 25 °C for 10 min [41]. The absorbance was measured at 420 nm and expressed in μM/g FW.

##### Catalase

The catalase (CAT) activity was conducted by mixing 478.5 μL of 25 mM potassium phosphate buffer containing in a final concentration 10 mM H_2_O_2_ and 12.5 μL of enzyme extract [42]. The CAT activity was determined by decomposing the H_2_O_2_ in 1 min at 240 nm and expressed as μM/g FW.

##### Superoxide Dismutase

The superoxide dismutase (SOD) enzyme determination method was conducted with minor modifications [43]. A mixture of 50 mM potassium dihydrogen phosphate (pH 7.8), 0.1 mM ethylenediaminetetraacetic acid, 75 mM nitro blue tetrazolium, 10 mM L-methionine, and 20 mM riboflavin were left to react with 100 µL of enzyme extract at room temperature under fluorescent lamps for 20 min before being placed in the dark. The activity of SOD was measured at 560 and expressed as mol/g FW.

### 2.4. Analysis of Defense-Related Genes

#### 2.4.1. Extraction of RNA and cDNA Synthesis

Three biological duplicates of each treatment’s leaves were collected at 15 dpi and stored at 80 °C until usage. A GeneJET RNA Purification Kit was used to isolate the total RNA (Thermo Fisher Scientific Co., Waltham, MA, USA). Each biological sample was made up of nine distinct samples from nine different plants. The purity of the isolated RNA was measured using UV–Vis spectroscopy (EMCLAB Instruments GmbH, Duisburg, Germany). Each sample was reverse transcribed to cDNA with GoScript™ Reverse Transcriptase (Promega, WI, USA) using a mix of oligo dT and hexamer random primers. In a gradient SureCycler (Agilent Technologies, Santa Clara, CA, USA), the RT-PCR reaction was carried out at 42 °C for 1 h and then deactivated at 80 °C for 5 min. The cDNA amplicons were stored at 20 °C until they were used in qRT-PCR.

#### 2.4.2. Quantitative Real-Time PCR (qRT-PCR) Assays

The accumulation levels of TMV coat protein gene (*TMV-CP*) as well as the transcriptional levels of three PR-proteins (*PR-1*, *PR-2*, and *PR-7*) and two phenylpropanoid pathway genes (*CHS* and *HQT*) were investigated. The primer sequences are presented in Table 1. The ratio of the expression of the TMV-CP gene and the housekeeping gene in tomato plants was used to calculate the viral accumulation level. The *β-actin* gene was used as a housekeeping gene to standardize the expression levels of all genes [44,45]. For the qRT-PCR assay, each biological treatment was carried out in three replicates using the GoTaq^®^ qPCR Master Mix (Promega, Wisconsin, USA) on PikoReal (Real-Time PCR, Thermo Fisher Scientific Co., Waltham, MA, USA) as previously reported [46]. The 2^−ΔΔCT^ method [47] was used to precisely quantify and calculate the relative transcriptional level of each tested gene.

### 2.5. Statistical Analysis

All data were statistically evaluated using the least significant difference (LSD) at a 0.05 probability using CoStat software (ANOVA). Gene expression (upregulation) was indicated by relative transcriptional levels greater than 1, whereas gene expression (downregulation) was indicated by values less than 1.

## 3. Results

### 3.1. Isolation and Identification of Trichoderma Isolate

The morphological analysis of the isolated *Trichoderma* isolate from tomato plant roots was compatible with the Trichoderma genus features according to the common taxonomic phenotypical criteria. Its conidia were green in color, single-celled, oval, smooth, or rough. Conidiophores were long, branching, and not whorly, with solo phialides or in-groups arising from tiny terminal bunches at a 90° angle from the conidiophore. A PCR technique was used to confirm the morphological identification of the *Trichoderma* isolate by amplifying PCR amplicons of three genes of *ITS*, *rpb2*, and *tef1* with approximately 600, 1050, and 500 bp, respectively.

**Figure 1:**
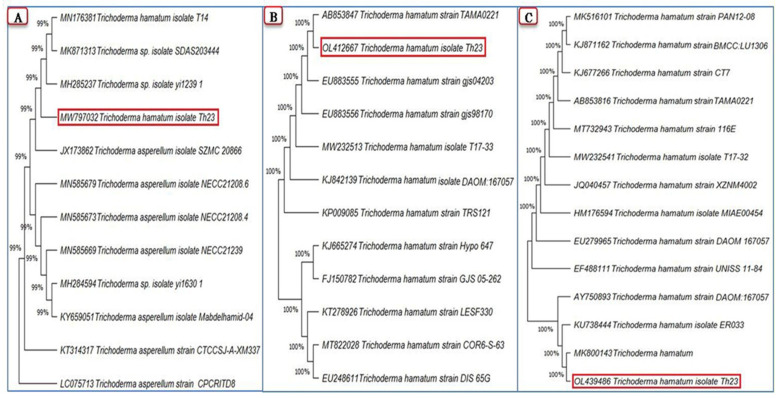
Phylogenetic trees show the relationship of the *Trichoderma hamatum* strain Th23 (shown in a red rectangle) among closely related *Trichoderma* isolates from GenBank based on partial sequences of three genes: *ITS* (**A**), *rpb2* (**B**), and *tef1* (**C**) generated by MEGA X software.

The partial sequences of the three amplified genes *ITS*, *rpb2*, and *tef1* were obtained, then submitted to NCBI GenBank, and were assigned to *Trichoderma hamatum* strain Th23 with accession numbers MW797032, OL412667, and OL439486, respectively. A comparison of the generated ITS region nucleotide sequence of the *T. hamatum* strain Th23 with the GenBank identified isolates demonstrated that the genetic homogeneity closest to 99% of the ITS sequence was with *Trichoderma* spp. (MH285237 and MK871313), *T. hamatum* (MN176381), and *T. asperellum* (JX173862) (Figure 1A). Comparing the *T. hamatum* strain Th23 *rpb2* nucleotide sequence with those isolates of *T. hamatum* in the GenBank database clarified that the highest homogeneity was 100% with *T. hamatum* (AB853847 and EU883555) (Figure 1B). The maximum nucleotide sequence similarity of the *tef1* gene (100%) was observed with *T. hamatum* isolates from different countries (MK800143, KU738444, and AY750893) (Figure 1C).

### 3.2. Effect of Th23 on Viral Symptoms Development, Tomato Plants Growth Parameters, and Total Chlorophyll Content

**Figure 2:**
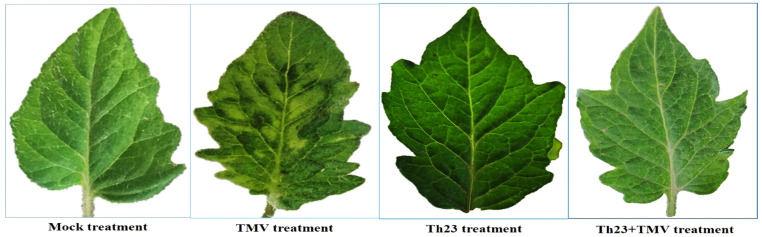
The protective activity of *Trichoderma hamatum* isolate Th23 against TMV on tomato leaves at 15 dpi (Th23 + TMV treatment). TMV treatment showed mild mosaic symptoms compared to Th23 and the mock treatments showed no developed symptoms.

Under greenhouse conditions, the protective activity of *Trichoderma hamatum* strain Th23 (anti-TMV) was evaluated on tomato plants. Compared to nontreated plants, Th23 + TMV treatment considerably reduced the disease symptoms and enhanced plant development. The TMV characteristic symptoms started to appear on tomato leaves of TMV-inoculated plants (TMV treatment) at 12 dpi and ended with a severe mosaic pattern and yellowing symptoms at 15 dpi (Figure 2). On the other hand, a five-day delay with mild mosaic symptom development was observed in the Th23 + TMV treatment tomato plants. No TMV signs were observed on tomato plants treated with Th23 or the mock (control) (Figure 2).

Compared to the mock and TMV treatments, the Th23 and Th23 + TMV treatments had a significant impact (*p* ≤ 0.05) on the shoot and root system characteristics (Table 2). For growth parameters, shoot, and root, the Th23 treatment exhibited the highest values, followed by the Th23 + TMV treatment. The Th23 treatment showed the highest shoot length (40.31 cm), fresh (11.33 g), and dry (4.08 g) weights, which recorded an increase in percentages of 74.35%, 91.06%, and 42.16%, respectively (Table 2). On the other hand, Th23 + TMV treatment exhibited significant increases of 50.22%, 27.15%, and 11.85% for shoot length, fresh, and dry weights, respectively (Table 2). Compared to the mock treatment, the tomato plants treated with TMV alone showed a considerable reduction in the shoot system’s length, fresh, and dry weights, recorded at 23.12 cm, 5.93 g, and 2.87 g, respectively. Similarly, significant (*p* ≤ 0.05) increases in length or fresh and dry weights of the root system by 95.08%, 94.75%, and 45.05% were recorded after treatment of the tomato plant with Th23 (Table 3). Moreover, Th23 + TMV treatment revealed significant increases in the length or fresh and dry weights of the root system of tomato plants, which reached 14.56 cm, 4.75 g, and 2.05 g with an increasing percentage of 49.18%, 55.74%, and 12.64%, respectively (Table 3). In contrast, significant reductions in the root system parameters were detected after infection with TMV in nontreated tomato plants (Table 3).

A significant difference in the total chlorophyll content of tomato leaves was observed in all treatments. The Th23 treatment exhibited the highest chlorophyll content (41.33 SPAD unit), followed by the mock treatment (35.89 SPAD unit), Th23 + TMV treatment (34.81 SPAD unit), and the TMV treatment (28.85 SPAD unit). Compared to the control (mock) treatment, the Th23 treatment showed a significant increase of 15.16%, while TMV treatment exhibited a reduction in total chlorophyll by 24.40% in tomato leaves suffering from higher levels of mosaic and disease symptoms.

### 3.3. Effect of Th23 Application on TMV Accumulation Level

Regarding the accumulation levels of TMV particles inside the infected tissues, the Th23 + TMV treatment plants exhibited a significant decrease in the accumulation levels of TMV when compared to the TMV treatment plants. The TMV content was quantified based on the ratio of the cycle threshold (Ct) value of the *TMV-CP* gene to the tomato internal control actin gene. The qRT-PCR data showed that the accumulation level of *TMV-CP* of the TMV treatment was a 29.32-fold change, whereas it was 4.49-fold for the Th23 + TMV treatment plants. No TMV was detected in the mock or Th23 tomato plant treatments. Thus, the small amount of TMV detected in the Th23 pretreated plants—with a considerable reduction in viral accumulation level by 84.69%—indicated that Th23 could induce plant resistance to TMV proliferation in tomato tissues.

### 3.4. Oxidative Stress Markers Assay

**Figure 3:**
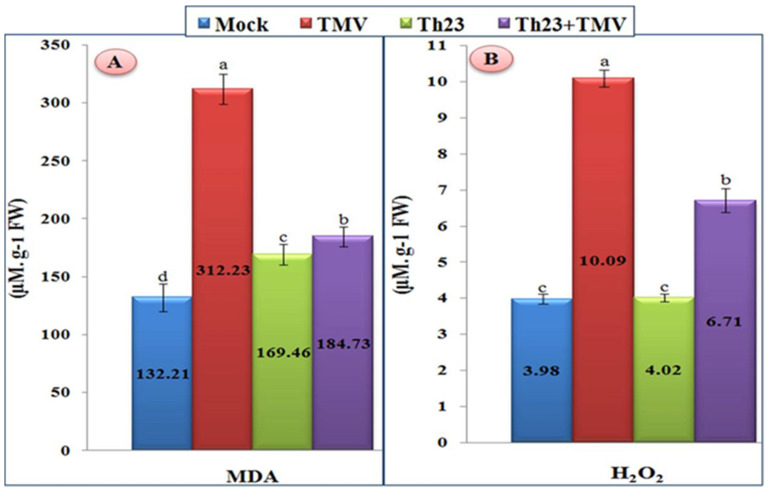
(**A**) Malondialdehyde content (MDA) and (**B**) hydrogen peroxide accumulation levels as affected by various treatments including untreated plants (mock), TMV-treated plants (TMV), *Trichoderma hamatum* strain Th23 application (Th23), and *T. hamatum* strain Th23 application 48 h before TMV inoculation (Th23 + TMV). The mean values in each of the columns that begin with the same letter are not statistically different (*p* ≤ 0.05).

Compared to mock treatment, the tomato plant leaves infected with TMV showed a significant increase in the content of H_2_O_2_ and MDA (Figure 3). For MDA, the TMV treatment exhibited the highest level (312.23 µM/g FW) with a significant increase of 136.16% compared to the mock treatment of 132.21 µM/g FW. On the other hand, the tomato plants of two treatments, Th23 and Th23 + TMV, showed a considerable reduction in MDA content compared to the TMV treatment. The Th23 treatment recorded 169.46 µM/g FW while Th23 + TMV showed 184.73 µM/g FW (Figure 3A). Similar to MDA, H_2_O_2_ was significantly elevated upon TMV infection (Figure 3B). The TMV treatment showed the greatest H_2_O_2_ level (10.09 µM/g FW), which exhibited a considerable increase of 153.52% compared to the control (3.98 µM/g FW). On the other hand, the pretreatment of tomato plants with Th23 (Th23 + TMV treatment) recorded 6.91 µM/g FW with significant decreases in the H_2_O_2_ level by 31.52% compared to the nontreated tomato plants. Moreover, no significant difference between the Th23 treatment (4.02 µM/g FW) and mock treatment plants was recorded (Figure 3B).

### 3.5. Antioxidant Enzymes Activity

**Figure 4:**
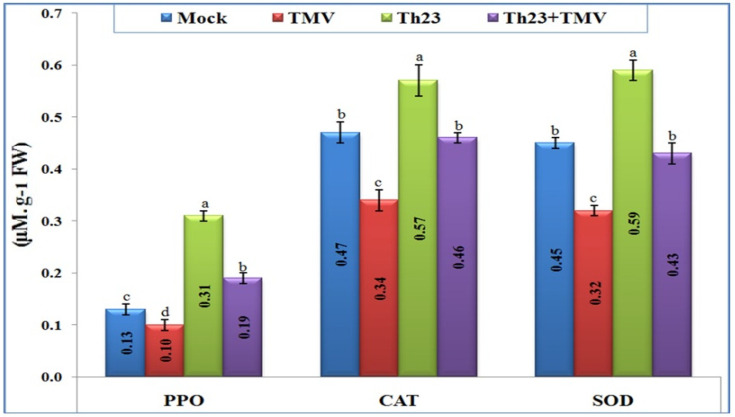
Polyphenol oxidase (PPO), catalase (CAT), and sodium dismutase (SOD) kinetic levels as affected by various treatments including untreated plants (mock), TMV-treated plants (TMV), *Trichoderma hamatum* strain Th23 application (Th23), and *T. hamatum* strain Th23 application 48 h before TMV inoculation (Th23 + TMV). The mean values in each of the columns that begin with the same letter are not statistically different (*p* ≤ 0.05).

The three antioxidant enzymes, catalase (CAT), polyphenol oxidase (PPO), and superoxide dismutase (SOD), were clearly differentiated upon TMV infection and Th23 treatments (Figure 4). Most importantly, Th23 treatment induced an antioxidant defense system and significantly increased the three-enzyme content inside tomato leaves. For PPO activity, the Th23 treatment exhibited the highest level (0.31 µM/g FW), with an increase in its activity of 138.46% and 210% when compared to the mock treatment (0.13 µM/g FW) and TMV treatment (0.10 µM/g FW), respectively. Similarly, the Th23 + TMV treatment induced the PPO activity (0.19 µM/g FW) with significant increases of 46.15% and 90% compared to the mock and TMV treatments, respectively (Figure 4). Regarding antioxidant enzyme CAT activity, the Th23-pretreated tomato plants showed the greatest level of content (0.57 µM/g FW) with a significant increase of 67.65% and 21.27% compared to TMV and mock treatments, respectively (Figure 4). In addition, Th23 + TMV treatment exhibited 0.46 µM/g FW activity with an increase of 35.29% compared to then nontreated plants. Compared to the mock treatment (0.47 µM/g FW), no significant change (*p* ≤ 0.05) was reported with Th23 + TMV treatment (Figure 4). Concerning SOD activity, the results showed that the SOD level content was considerably decreased upon TMV infection. The TMV treatment was significantly reduced by 27.88% compared to the control (0.45 µM/g FW). Although there was a slight reduction in SOD activity of Th23 + TMV treatment (0.43 µM/g FW), no significant difference was reported compared with the control. The Th23 treatment showed the highest SOD activity (0.59 µM/g FW) with significant increases of 84.38% and 31.11% compared to the TMV and mock treatments, respectively (Figure 4).

### 3.6. Transcriptional Levels of Defense-Related Genes

#### 3.6.1. Polyphenolic Biosynthetic Pathway

**Figure 5:**
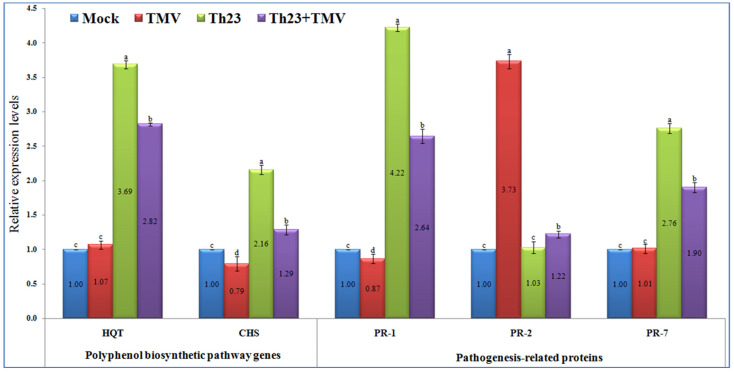
Polyphenol biosynthetic pathway genes (*HQT* and *CHS*) and pathogenesis-related proteins’ (*PR-1*, *PR-2*, and *PR-7*) relative expression levels quantified by qRT-PCR as affected by various treatments including untreated plants (mock), TMV-treated plants (TMV), *Trichoderma hamatum* strain Th23 application (Th23), and *T. hamatum* strain Th23 application 48 h before TMV inoculation (Th23 + TMV). The mean values in each of the columns that begin with the same letter are not statistically different (*p* ≤ 0.05).

The qRT-PCR results showed significant increases (*p* ≤ 0.05) in the relative expression levels of the two polyphenolic biosynthetic pathway genes, *HQT* and *CHS*, in the Th23-treated tomato plants, either challenged with TMV or not (Figure 5). The Th23 treatment recorded the highest transcript of *HQT* with a relative transcriptional level of 3.69-fold change higher than the mock treatment plants. Similarly, a significant increase with a relative transcriptional level of 2.82-fold change greater than the control was reported in the Th23 + TMV treatment tomato plants. No significant difference was noted between the TMV treatment and control (Figure 5). Regarding the *CHS* transcript, it was reported that TMV treatment suppressed and significantly downregulated *CHS* in tomato plant tissues. Intriguingly, the application of Th23 induced *CHS* transcripts in Th23 and Th23 + TMV treatments. The Th23 treatment exhibited the highest relative transcriptional level (2.16-fold), followed by the Th23 + TMV treatment with a 1.29-fold change higher than the mock treatment. The TMV treatment showed a relative expression level of 0.79-fold lower than mock treatment plants (Figure 5).

#### 3.6.2. Pathogenesis-Related Proteins

In the present study, the three pathogenesis-related proteins (*PR-1*, *PR-2*, and *PR-7*) were significantly differentiated upon challenging tomato plants with Th23 and/or TMV infection (Figure 5). For *PR-1*, it was shown that the treatment of tomato plants with Th23 isolate, either alone or before TMV infection, triggered the expression of the *PR-1* gene. The Th23 treatment showed a relative expression level of 4.22-fold change higher than the control, which revealed a significant increase of 385.06% compared to the TMV treatment. Furthermore, Th23 + TMV treatment induced *PR-1* with a relative expression level of 2.64-fold change higher than the mock treatment. The nontreated tomato plants challenged with TMV only exhibited a significant (*p* ≤ 0.05) decreasing *PR-1* level, with a relative expression level of 0.87-fold change lower than the control (Figure 5). Regarding the *PR-2* transcript profile, it was reported that the TMV infection considerably induced *PR-2* in the infected tomato tissues with a relative expression level of 3.73-fold higher than the mock treatment. The Th23 + TMV treatment exhibited a slight upregulation with a relative transcriptional level of 1.22-fold higher than the control. Among the Th23 and mock treatments, no significant change was reported (Figure 5). Concerning *PR-7*, the treatment of tomato plants with the Th23 isolate significantly induced the expression of *PR-7*. The Th23 treatment exhibited the highest expression level (2.76-fold), while the Th23 + TMV treatment showed a relative transcriptional level of 1.90-fold higher than the mock treatment. On the other hand, no significant difference was observed in the TMV treatment compared to the mock treatment at *p* ≤ 0.05 (Figure 5).

## 4. Discussion

Plant diseases, particularly plant viral infestations, are responsible for significant crop losses, and pose a serious threat to food security all over the world [48]. Due to the difficult problem of their control and their changing environmental conditions, it is urgently necessary to discover and identify new biocontrol agents capable of controlling plant viral infections. The use of plant growth-promoting microorganisms as biocontrol agents is a safe alternative to the harmful use of chemicals and is seen as a long-term and environmentally friendly alternative [45]. There are, to date, few studies on the role of *Trichoderma* spp. in the induction of plant defenses against viral infections. The majority of prior research has spotlighted the critical role of *Trichoderma* spp. in the management of plant-fungal and bacterial diseases. Due to the increasing number of species and the paucity of morphological characteristics, it is very difficult to differentiate *Trichoderma* species using morphological characteristics [49,50]. Sequences of the most variable regions of specific genes are now becoming increasingly useful in identifying closely related species. In the current study, the *ITS* (Acc# MW797032) sequencing BLAST and phylogenetic tree results were not sufficient for species delimitation of the isolated *Trichoderma* strain. It was reported that the analysis based on *ITS* sequences has been shown to be ineffective in distinguishing closely related species within *Trichoderma* species complexes [51]. The gene sequences of *tef1* and *rpb2* genes are very informative and have been shown to be useful in investigating closely related strains at the species level [50,52]. As a result, the *tef1* and *rpb2* genes have become the preferred markers for identifying *Trichoderma* strains [53]. Based on the NCBI-BLAST alignment and phylogenetic tree analysis of *rpb2* (Acc# OL412667) and *tef1* (Acc# OL439486) genes, the isolated *Trichoderma* strain was identified as *Trichoderma hamatum* strain Th23.

Under greenhouse conditions, the soil application of the Th23 isolate, either alone or 48 h before TMV infection, significantly (*p* ≤ 0.05) enhanced the growth of tomato plants as well as chlorophyll content. At 15 dpi, the Th23 treatment exhibited the highest growth parameter values, followed by the Th23 + TMV treatment. On the other hand, the TMV treatment showed a significantly negative impact on the growth of tomato plants at all the growth parameters. Several authors have shown that the application of *Trichoderma* spp. was associated with an increase in root length, shoot length, and dry weight compared to the control plants [9,54,55,56]. Generally, the severe morphological and physiological changes including mosaic symptoms that occur upon viral infection are linked to changes in chlorophyll content and result in reduced photosynthesis [21,57]. Thus, the changes in chlorophyll pigment content upon viral infection can be used to indicate the functional status of photosynthesis in plants [58]. In the current study, the analysis of chlorophyll content showed that Th23 and Th23 + TMV treatments resulted in significant changes in chlorophyll content compared to tomato leaves of the TMV treatment. These results were in agreement with the findings of other researchers who showed that the application of *Trichoderma* spp. increased the chlorophyll content of the treated plants [59,60], while chlorophyll reduction was associated with a viral infection [57,58]. Consequently, the application of Th23 strain can boost photosynthetic rates and efficiency in plants [10], primarily through enhancing the plant’s redox state [61]. The current findings confirmed that Th23 can protect tomato plants against TMV not only by modulating symptoms and thus reducing disease severity, but also by lowering viral accumulation inside plant tissues. The observation of symptoms as well as the severity of the disease demonstrated that Th23 treatment reduced TMV in all treated plants. On the other hand, the significant decrease in TMV accumulation level (84.69%) confirmed the protective efficacy of Th23 against TMV infestation. Thus, the obtained data suggest that Th23 could stimulate the host’s innate immune system and/or trigger SAR, resulting in TMV suppression and/or replication inhibition. The findings were consistent with those of Tamandegani et al. [57], who reported that the pretreatment of soils with *T. asperellum* was associated with a significant reduction in CMV accumulation levels when compared with the untreated control cucumber plants. It was reported that Trichoderma-induced resistance to viral infection was usually associated with a reduction in virus concentration and disease severity, indicating that different defensive pathways were implicated [6,57,62]. The obtained results were consistent with the reported protective activities of different *Trichoderma* spp. against various plant viral infestations. It was shown that pretreating plants with *Trichoderma* spp. resulted in a considerable reduction in viral accumulation compared to nontreated plants [15,57,62].

Oxidative burst accumulation is one of the first reactions in the plant defense system. Reactive oxygen species (ROS) regulate various cellular processes including antimicrobial activity and the regulation of certain plant transcription factors [63]. One of the ROS molecules belonging to nonradical oxidants is hydrogen peroxide (H_2_O_2_). In plant–pathogen interactions, H_2_O_2_ serves various functions including inhibition of pathogen propagation, affects the defense system, and is a SAR signal molecule [64]. In the current study, H_2_O_2_ levels in the TMV treatment were 2.53 times higher than in the mock treatment plants. The same findings were obtained in a study on CMV reported by Song et al. [65], who noticed CMV enhanced H_2_O_2_ buildup in tomato and cucumber chloroplasts and mitochondria. Additionally, a study by Sorahinobar et al. [66] reported that H_2_O_2_ builds up fast once the virus infects the host, increasing resistance. At the same time, H_2_O_2_ levels were decreased in the Th23 + TMV treatment compared to the TMV treatment. This decrease may have been due to the protective activity of Th23 and a reduction in the TMV accumulation inside the treated tomato tissues. This result was similar to the findings of Luo et al. [67], who noticed that CMV + *T. asperellum* decreased the damaging effects of ROS in cucumber; thus, one probable mechanism of *T. asperellum*-induced CMV resistance is the stimulation of early plant defense mechanisms. Regarding the MDA level, the TMV treatment showed 2.36 times higher MDA levels compared to the controls. MDA accumulation had a limited significant increase in Th23 and Th23 + TMV treatments (169.46 and 184.73 µM/g FW, respectively) compared to the control (132.21 µM/g FW). These findings were consistent with those of Sobhy et al. [68] and Loreto and Velikova [69]. They suggested that an increase in MDA could indicate that plants are under oxidative stress, and thus could be a promising biomarker of membrane breakdown in pathogen-infected plants. Similar results were reported by Anthony et al. [70] in bananas infected with *Fusarium* fungus.

It is well known that CAT protects plant cells under stress exposure from ROS oxidative damage by converting ROS components to less toxic and more stable molecules such as oxygen and water [71,72]. In our study, the CAT enzyme decreased in tomato plants inoculated with TMV compared with the control, while it significantly increased in tomato plants treated with Th23 alone and Th23 + TMV. It seems that the preapplication of Th23 before TMV inoculation normalizes and cures the plant from TMV infection and keeps the accumulation levels of the CAT enzyme in normal conditions compared with the control plants. It has been proposed that CAT increases cell wall resistance, induces defense genes, accumulates SA signal, and suppresses RNA silencing [73]. The CAT enzyme may interact with viral movement protein, which is involved in symptom induction such as the CMV 2b protein reported by Mathioudakis et al. [74]. Likewise, the enhanced synthesis of antioxidant enzymes such as SOD and PPO in infected tomato plant leaves counteracted the higher MDA and H_2_O_2_ levels, limiting tissue oxidation [72,75] as well as limiting pathogen invasion by reinforcing cell walls [76]. Such antioxidant enzymes were significantly higher in plants that interacted with pathogens following exposure to the biocontrol agent such as *Trichoderma* spp., which triggers the induced systematic resistance by secreting defense-related enzymes including PPO, against viral pathogens, according to previous studies [57,77,78]. In our investigation, tomato plant inoculation with TMV alone caused a significant decrease in PPO and SOD activity. In contrast, in plants treated with Th23 only or with Th23 + TMV, both PPO and SOD enzyme activities reached their maximum levels. This change suggested that these enzymes could play a crucial role in ROS detoxification. Thus, the Th23 strain could activate PPO and SOD to prevent the tomato plant cells from TMV multiplication and transmission by establishing polymerized phenolic barriers around infection sites to kill the pathogen [79,80].

The expression of hundreds of genes is triggered by the plant–virus interaction. It was reported that plant defensive responses were regulated by crosstalk between the SA, JA, and ET signaling pathways [81].

The qRT-PCR results showed that the relative expression levels of the two polyphenolic biosynthesis pathway genes, *HQT* and *CHS*, were significantly increased in Th23-treated tomato plants, whether challenged with TMV or not. *CHS*, the first enzyme in the flavonoid pathway, transforms *p*-coumaroyl CoA into naringenin chalcones and is regarded as a key precursor necessary for plant flavonoids [35,44]. *HQT* is a principal enzyme in the biosynthesis of chlorogenic acid, catalyzing the conversion of caffeoyl-CoA and quinic acid to chlorogenic acid [82]. Chlorogenic acid is a polyphenolic compound that is important in improving plant resistance and inhibiting pathogens such as viruses [83,84]. In the current investigation, the Th23 treatment had the greatest transcript of *HQT* with a relative transcriptional level 3.69-fold greater than the mock-treated plants. Similarly, with the Th23 + TMV treatment, tomato plants showed a substantial rise, with a relative expression level 2.82-fold higher than the control. In terms of the *CHS* transcript, it has been demonstrated that TMV infection significantly decreased *CHS* expression in tomato plant tissues after TMV treatment. Surprisingly, the application of Th23 induced *CHS* transcripts in Th23 and Th23 + TMV treatments. It was reported that the roots invaded by *T. harzianum* demonstrated great resistance against dangerous organisms, which was connected with changes in phenolic accumulation by Yedidia et al. [85]. Consequently, phenolics accumulating in *Trichoderma*-treated plants can act as electron and hydrogen donors, protecting plant tissue from oxidative damage during pathogen infection [86]. Previous research has found that overexpression of *CHS* could result in a large accumulation of flavonoid and isoflavonoid compounds with broad antimicrobial activity against a wide range of phytopathogens [87,88,89,90]. Additionally, it was reported that increases in chlorogenic acid levels were linked to increased *HQT* expression and vice versa [82,88]. Thus, the elevation of transcriptional expression of these genes demonstrates their antiviral role, implying that the tomato plant can utilize polyphenolic compounds as one of its defenses against viral infection and spread. In line with the obtained results, the increased expression levels of *CHS* and *HQT* resulted in the accumulation of polyphenolic compounds inside plant tissues, the development of SAR, and increased resistance against TMV infestation [21]. As a result, pretreating tomato plants with Th23 may result in an increase in numerous flavonoid compounds. Thus, Th23 might be used to combat TMV infections as a biocontrol agent. However, further study is needed for future field uses.

Several reports have proposed that a convergent collection of PR proteins is responsible for SAR development and is effective in suppressing pathogen multiplication and/or dissemination [21,91,92]. For *PR-1*, it was shown that the TMV treatment exhibited a significant decrease in *PR-1* expression level by 0.77-fold lower than the control at *p* ≤ 0.05. On the other hand, the Th23 treatment or Th23 + TMV treatment triggered the expression level of *PR-1* with significant increases of 385.06% and 203.45%, respectively, when compared with the TMV treatment. Salicylic acid (SA) is a well-known plant signal phytohormone molecule, and its role in plant immune activation has been documented for more than two decades [20,93,94]. Furthermore, several studies have shown that *PR-1* is an SA marker gene, an important regulator of SAR, and a predictor of early plant defensive response [93,95]. Meanwhile, induction of *PR-1* is frequently associated with SA accumulation, which results in SAR activation [20,96,97]. As a result, we hypothesized that Th23 may produce elicitor metabolite compounds that induce systemic resistance, activating SAR, and enhancing plant resistance to viral infection. *PR-7*, the most prominent *PR* gene in tomato plant tissues, encodes endoproteinase activity in plants. It has emerged as a major component of plant defensive response proteins against various pathogens [91,98,99,100]. In this study, the Th23 treatment had the highest expression level, followed by Th23 + TMV treatment, with a relative transcriptional level 2.76- and 1.90-fold higher than the mock treatment, respectively. It was postulated that *PR-7* participated in pathogen perception and signaling cascade activation in infected tomato plants via specialized processing of a *LRP* (leucine-rich repeat protein) [101,102]. However, more characterization and functional analysis of *PR-7* will lead to a more in-depth understanding of its role in plant–viral interactions.

In addition to their primary role in viral transport from cell to cell, *PR-2* encoding -1,3-glucanases mediate cell-to-cell communication and long-distance signaling by limiting callose deposition near plasmodesmata [103,104,105]. They are involved in pathogenic defense and several physiological plant activities and are primarily induced by SAR and SAR inducers such as SA [37,106,107]. In the current study, TMV infection considerably induced *PR-2* in the TMV treatment with a relative transcriptional level 3.73-fold higher than in the mock treatment. The findings were consistent with previous research that revealed significant activation of *PR-2* in response to viral infections in a variety of plant species including *Arabidopsis*, tobacco, potato, and tomato [37,108,109,110,111]. Moreover, a lack of tobacco *PR-2* expression was associated with lower viral infection susceptibility [111], whereas overexpression accelerated PVY infection spread across cells [112,113]. Interestingly, treatments of tomato plants with Th23 prior to TMV infection showed a slight increase in *PR-2* compared to the control plants. There were no significant changes reported between Th23 and mock treatments in tomato plants at *p* ≤ 0.05. Thus, the preapplication of Th23 before viral infection may reduce TMV infection by lowering *PR-2* expression and inhibiting long-distance viral movement between cells.

## 5. Conclusions

In this study, we investigated, for the first time, the effects of *Trichoderma hamatum* Th23 in tomato plants as a safeguard against TMV. Th23 can induce tomato systemic resistance against TMV by modulating the plant response, triggering multiple plant defense pathways, increasing resistance, and preventing the suppression of defense genes. The preapplication of Th23 before TMV infection significantly enhanced tomato growth parameter heights, improved total chlorophyll, decreased disease severity, and reduced TMV accumulation inside infected tissues. Moreover, a reduction in oxidative stress markers (MDA and H_2_O_2_) and elevation of the antioxidant enzymes (SOD, CAT, and PPO) were also reported. In addition, the triggering of transcriptional levels of *HQT*, *CHS*, *PR-1*, and *PR-7* was observed. The results implied the potential of Th23 application in plant viral disease control.

## Figures and Tables

**Table 1:** Sequences of primers used in this study.

Primer Code	Target Gene	Direction	Nucleotide Sequences (5′-3′)	References
*ITS*	*Internal Transcribed Spacer*	Forward	TCCGTAGGTGAACCTGCGG	[30]
		Reverse	TCCTCCGCTTATTGATATGC	
*rpb2*	*RNA polymerase II subunit 2*	Forward	GAYGAYMGWGATCAYTTYGG	[31]
		Reverse	CCCATRGCTTGYTTRCCCAT	
*tef1*	*Translation elongation factor 1 alpha*	Forward	CATCGAGAAGTTCGAGAAGG	[32]
		Reverse	AACTTGCAGGCAATGTGG	
*TMV-CP*	*Tobacco mosaic virus-coat protein*	Forward	ACGACTGCCGAAACGTTAGA	[33]
		Reverse	CAAGTTGCAGGACCAGAGGT	
*PR-1*	*Pathogenesis related protein-1*	Forward	CCAAGACTATCTTGCGGTTC	[34]
		Reverse	GAACCTAAGCCACGATACCA	
*PR-2*	*β-1,3-glucanases*	Forward	TATAGCCGTTGGAAACGAAG	[34]
		Reverse	CAACTTGCCATCACATTCTG	
*PR-7*	*Proteinase*	Forward	AACTGCAGAACAAGTGAAGG	[34]
		Reverse	AACGTGATTGTAGCAACAGG	
*CHS*	*Chalcone synthase*	Forward	CACCGTGGAGGAGTATCGTAAGGC	[35]
		Reverse	TGATCAACACAGTTGGAAGGCG	
*HQT*	*Hydroxycinnamoyl Co A: quinate hydroxycinnamoyl transferase*	Forward	CCCAATGGCTGGAAGATTAGCTA	[35]
		Reverse	CATGAATCACTTTCAGCCTCAACAA	
*β-actin*	*Beta-actin*	Forward	ATGCCATTCTCCGTCTTGACTTG	[36]
		Reverse	GAGTTGTATGTAGTCTCGTGGATT	

**Table 2:** The effect of different treatments of Th23 and TMV inoculation on the length, fresh, and dry weights of the shoot system in tomato plants.

Treatment	Shoot ± SD *					
	Length (cm)	Increase (%)	Fresh Weight (g)	Increase (%)	Dry Weight (g)	Increase (%)
Mock	33.44 ± 3.29 c	44.64	7.59 ± 2.01 b	27.99	3.23 ± 0.51 b	12.54
TMV	23.12 ± 5.21 d	-----	5.93 ± 2.48 c	-----	2.87 ± 0.64 c	-----
Th23	40.31 ± 3.34 a	74.35	11.33 ± 3.97 a	91.06	4.08 ± 0.41 a	42.16
Th23 + TMV	34.73 ± 3.98 b	50.22	7.54 ± 1.98 b	27.15	3.21 ± 0.35 b	11.85

* SD, Standard deviation. The mean values in each of the columns that begin with the same letter are not statistically different (*p* ≤ 0.05).

**Table 3:** The effect of different treatments of Th23 and TMV inoculation on the length, fresh, and dry weights of the root system in tomato plants.

Treatment	Root ± SD *					
	Length (cm)	Increase (%)	Fresh Weight (g)	Increase (%)	Dry Weight (g)	Increase (%)
Mock	11.41 ± 0.63 c	16.91	4.11 ± 0.45 c	34.75	2.03 ± 0.63 b	11.54
TMV	9.76 ± 0.72 d	-----	3.05 ± 0.34 d	-----	1.82 ± 0.47 c	-----
Th23	19.04 ± 2.14 a	95.08	5.94 ± 1.19 a	94.75	2.64 ± 0.51 a	45.05
Th23 + TMV	14.56 ± 2.18 b	49.18	4.75 ± 1.23 b	55.74	2.05 ± 0.72 b	12.64

* SD, Standard deviation. The mean values in each of the columns that begin with the same letter are not statistically different (*p* ≤ 0.05).

## Data Availability

All data reported here are available from the authors upon request.
